# Predicting hospital-acquired pneumonia among schizophrenic patients: a machine learning approach

**DOI:** 10.1186/s12911-019-0792-1

**Published:** 2019-03-13

**Authors:** Kuang Ming Kuo, Paul C. Talley, Chi Hsien Huang, Liang Chih Cheng

**Affiliations:** 1 Department of Healthcare Administration, I-Shou University, No.8, Yida Rd., Yanchao District, Kaohsiung City, 82445 Taiwan, ROC; 2 Department of Applied English, I-Shou University, No. 1, Sec. 1, Syuecheng Rd., Dashu District, Kaohsiung City, 84001 Taiwan, ROC; 3 Department of Community Healthcare & Geriatrics, Nagoya University Graduate School of Medicine, Nagoya, Japan; 4 Department of Family Medicine, E-Da Hospital, Kaohsiung City, Taiwan, ROC; 5 Center for Evidence-based Medicine, E-Da Hospital, Kaohsiung City, Taiwan, ROC; 6 School of Medicine for International Students, I-Shou University, Kaohsiung City, Taiwan, ROC; 7 Department of Pharmacy, Yo-Chin Hospital, Kaohsiung City, Taiwan, ROC

**Keywords:** Clozapine, Machine learning, Pneumonia, Risk factors, Schizophrenia

## Abstract

**Background:**

Medications are frequently used for treating schizophrenia, however, anti-psychotic drug use is known to lead to cases of pneumonia. The purpose of our study is to build a model for predicting hospital-acquired pneumonia among schizophrenic patients by adopting machine learning techniques.

**Methods:**

Data related to a total of 185 schizophrenic in-patients at a Taiwanese district mental hospital diagnosed with pneumonia between 2013 ~ 2018 were gathered. Eleven predictors, including gender, age, clozapine use, drug-drug interaction, dosage, duration of medication, coughing, change of leukocyte count, change of neutrophil count, change of blood sugar level, change of body weight, were used to predict the onset of pneumonia. Seven machine learning algorithms, including classification and regression tree, decision tree, k-nearest neighbors, naïve Bayes, random forest, support vector machine, and logistic regression were utilized to build predictive models used in this study. Accuracy, area under receiver operating characteristic curve, sensitivity, specificity, and kappa were used to measure overall model performance.

**Results:**

Among the seven adopted machine learning algorithms, random forest and decision tree exhibited the optimal predictive accuracy versus the remaining algorithms. Further, six most important risk factors, including, dosage, clozapine use, duration of medication, change of neutrophil count, change of leukocyte count, and drug-drug interaction, were also identified.

**Conclusions:**

Although schizophrenic patients remain susceptible to the threat of pneumonia whenever treated with anti-psychotic drugs, our predictive model may serve as a useful support tool for physicians treating such patients.

## Background

It is commonly recognized that pneumonia can increase medical costs and is a particular burden since these patients must significantly utilize medical resources and services [1]. Further, evidence reveals that pneumonia-related deaths are more prevalent when compared with pneumonia-unrelated deaths [2]. It is therefore crucial to plan and prepare some form of pneumonia prevention effort in order to better diminish the occurrence of pneumonia among at-risk patients.

On the other hand, schizophrenia, a severe mental disorder that influences more than 21 million people worldwide [3], can also impose a similar considerable burden on medical expenses [4]. Schizophrenic patients are reported to be more likely to die early than the general public due to preventable diseases related to cardiovascular disease, metabolic disease and infections [3]. Schizophrenia can be treated with medications and psychological support [3]; evidence [5, 6], however, reports that anti-psychotic medicine is effective but may lead to cases of pneumonia. Considering that people with schizophrenia are usually vulnerable and may face discrimination or violation of their basic human rights [3], the very real question of how to prevent fatal diseases such as pneumonia that often accompanies their treatment of schizophrenia is therefore a pressing issue that should not be neglected.

Many scholars [5, 7, 8] have investigated issues related to hospital-acquired pneumonia by traditional statistical models and surely advance our knowledge of pneumonia. Later studies [9–11] further utilized machine learning approaches to investigate pneumonia-related issues. Among those studies, little evidence however utilized machine learning techniques to predict the risk factors of pneumonia specifically among schizophrenic patients. Without deeper knowledge of how schizophrenic patients develop hospital-acquired pneumonia, comprehensive preventative strategies cannot be possibly formulated to counter this serious threat. The study purpose is to build a predictive model for hospital-acquired pneumonia among schizophrenic patients by adopting machine learning techniques. Since machine learning techniques are able to analyze data unsuitable for use in traditional statistical models, different perspectives can be acquired and those findings can further provide support to healthcare professionals’ clinical decision-making.

## Related work

To date, various studies have analyzed risk factors of contracting pneumonia based on traditional statistical models which require strict assumptions. Their findings revealed that multiple risk factors can influence the occurrence of pneumonia among patients. For example, Mortensen, Coley, Singer, Marrie, Obrosky, Kapoor and Fine [2] reported that leukopenia was one of the factors that associated with the mortality of pneumonia. Manabe, Teramoto, Tamiya, Okochi and Hizawa [8] concluded that sputum suctioning, deterioration of the swallowing function, dehydration, and dementia were all risk factors associated with aspiration pneumonia. Gupta, Boville, Blanton, Lukasiewicz, Wincek, Bai and Forbes [7] identified that mechanical ventilation patients have an increased risk of mortality. Regarding pneumonia-related studies that employed schizophrenic patients as their subjects, despite being efficacious medication for treating schizophrenia, anti-psychotic drugs may however cause unanticipated side-effects for schizophrenic patients. As example, several previous studies have found that anti-psychotic drugs can lead to the development of pneumonia [5, 12]. Further, drug-drug interaction between anti-psychotic drugs and anxiolytic or anti-convulsive drugs could probably accelerate the occurrence of the pneumonia [13]. Evidence [6, 14] even showed that community-acquired pneumonia was associated with taking anti-psychotic drugs in elderly patients. Women were more likely to have a recurrence of pneumonia than men. The potential transmission mechanism underlying the influence of anti-psychotics remained unclear, but cardiopulmonary [15], agranulocytosis [16], and abnormal glucose regulation [17], are reported.

Moreover, Kuo, Yang, Liao, Chen, Lee, Shau, Chang, Tsai and Chen [5] reported although an increased risk of pneumonia was detected among the use of available anti-psychotics, only clozapine was associated with a dose-dependent increase. Therefore, use and titration of clozapine possesses a higher threat to patients with long-term management of schizophrenia.

Recently, a number of studies have adopted machine learning techniques to predict various issues concerning pneumonia. For example, Cooper, Aliferis, Ambrosino, Aronis, Buchanan, Caruana, Fine, Glymour, Gordon, Hanusa, et al. [9] applied eight machine learning methods to predict the mortality of inpatients with pneumonia. They found that neural network, hierarchical mixtures of experts, and logistic regression can attain the lowest error rate. Chapman, Fizman, Chapman and Haug [18] adopted machine learning algorithms including expert-rules, Bayesian network, and decision tree to identify onset pneumonia from thoracic X-ray reports. The performance of three algorithms differs in sensitivity, specificity, and precision; but, it is similar to physicians’ practice. Heckerling, Gerber, Tape and Wigton [10] integrated neural networks and genetic algorithms for predicting community-acquired pneumonia, and found that inclusion of genetic algorithms can help optimize neural networks algorithms. Caruana, Lou, Gehrke, Koch, Sturm and Elhadad [19] utilized high-performance, generalized additive models with pairwise interactions to predict the probability of death due to pneumonia. The results reveal that their proposed algorithm outperforms other algorithms such as logistic regression, random forest, and logitboost. Kim, Diggans, Pankratz, Huang, Pagan, Sindy, Tom, Anderson, Choi, Lynch, et al. [11] developed a machine learning model to classify usual interstitial pneumonia patients, and concludes that their model is feasible for predicting usual interstitial pneumonia occurrence.

A review of the literature reveals a clear gap regarding pneumonia-related studies. Despite a great deal of previous research having been focused on the risk factors of or outcome of pneumonia [2, 7, 8], less research utilizing machine learning techniques was carried out specifically related to schizophrenic patients. Due to the special characteristics and possible influences of schizophrenia on patients’ health conditions, it is therefore imperative to develop a predictive model for risk factors associated with pneumonia. Such a model can be based on machine learning techniques which can analyze health data while even successfully violating statistical assumptions.

## Methods

### Data

Research data were obtained via a review of medical records taken from a 200-beds Taiwanese mental hospital. The majority of medical records are still paper-based since the subject hospital has only implemented a small-scale computerized physician order entry (CPOE) system to-date. Important variables related to the onset of pneumonia may not be as comprehensive as what the electronic medical record systems can provide. Following the procedures of medical record review suggested by literature [20, 21], we first trained one of our authors to be a qualified abstractor, and then developed a data abstraction instrument for purposes of abstracting data. Afterwards, we formulated protocols and guidelines for abstraction, and the abstraction accuracy was finally checked by an experienced staff member of the medical records department in order to ensure the reliability of the abstracted data.

Eligibility criteria were that a patient must (1) be diagnosed through an international classification of diseases, ninth revision, clinical modification (ICD-9-CM) starting with 295, or with ICD-10-CM starting with F20, (2) have been hospitalized, (3) aged over 20, and (4) have been recognized as an infection case between 2013 and 2018 by the infectious control committee of the subject hospital. We reviewed medical records and collected required data according to a patient list provided by the infectious control committee of the subject hospital.

Each patient was classified based on whether he/she had or had not acquired pneumonia during hospitalization (i.e., dependent variable). The medical records taken from infectious inpatients, excluding in-patients who were admitted to hospital due to their contracting pneumonia, were sent out for the pulmonologists’ diagnosis since the subject hospital operates only as a specialty mental hospital. If the patient was diagnosed with pneumonia, the dependent variable of that patient record would then be labeled as ‘Yes.’ Further, each patient contained eleven predictors including demographic information (i.e., gender, age, and change of weight), and blood test data (i.e., change of leukocyte count, change of neutrophil count, change of blood sugar level), medication information (i.e., clozapine use, drug-drug interaction, coughing, dosage, and duration of medication). Among the eleven predictors, a change in body weight, the change of one’s leukocyte/ neutrophil count, and the change of blood sugar level are deemed continuous, and measure the difference of body weight, leukocyte/neutrophil count, and blood sugar level between the onset of hospitalization and report as infection cases. Further, clozapine use, drug-drug interaction, and coughing are used as discrete variables, and indicate whether the patients have taken clozapine, have taken clozapine with fluoxetine/carpine/depatec simultaneously, and have coughs, respectively. Taking clozapine and fluoxetine/carpine/depatec concurrently may introduce drug-drug interaction and may accelerate the occurrence of pneumonia [13]. Finally, dosage and duration of medication are continuous variables, and they measure the total quantity of and total number of days of clozapine use by patients. An increased risk of pneumonia with a dose-dependent increase of clozapine was confirmed [5].

The selection of eleven predictors is primarily based on pneumonia-related literature [2, 5, 8, 12, 13], the suggestions of one physician specializing in infectious control measures, and also the availability of data taken from medical records. Totally, 185 eligible cases without missing values were collected. Table 1 showed the detailed operational definition of variables used in our study.

**Table 1:** Operational definition of variables

Variables		Measurement	Definition
Dependent	Pneumonia	Discrete	Does the infection patient acquire pneumonia during hospitalization? Yes or No.
Independent	Gender	Discrete	Gender of the patients, Male or Female.
	Age	Continuous	Age (in years) during hospitalization
	Clozapine use	Discrete	Have taken clozapine? Yes or No
	Drug-drug interaction	Discrete	Has the patient taken Clozapine with Fluoxetine/Carpine/Depatec at the same time?
	Coughing	Discrete	Does the patient have coughs? Yes or No.
	Dosage	Continuous	The amount of medication taken by patients.
	Duration of medication	Continuous	The total days patients took Clozapine.
	Change of leukocyte count	Continuous	The difference of leukocyte count between start hospitalization and reported as infection.
	Change of neutrophil count	Continuous	The difference of neutrophil count between start hospitalization and reported as infection.
	Change of blood sugar level	Continuous	The difference of blood sugar between start hospitalization and reported as infection.
	Change of weight	Continuous	The difference of body weight between start hospitalization and reported as infection.

### Experimental setup

In order to predict risk factors for schizophrenia inpatients, we adopted R 3.5.1 [22], an open source statistical platform for data analysis. Prior pneumonia- related studies have adopted several machine-learning algorithms such as decision tree [23], k-nearest neighbors [24], naïve Bayes [25], random forest [19], logistic regression [19], and support vector machine [25, 26]. We therefore chose seven algorithms, including classification and regression tree (CART), decision tree (C5.0), k-nearest neighbors (KNN), naïve Bayes (NB), random forest (RF), support vector machine (SVM), and logistic regression (LGR), to build the predictive model and also to compare the performance of those machine learning algorithms along with prior studies. Further, we adopted the caret 6.0–80 package [27] to automatically tune the optimal combinations of model parameters (*see* Table 2) for the seven machine learning algorithms aiming to achieve a better prediction performance.

**Table 2:** Model parameter settings

Method	Parameter	Best parameter setting
CART	cp	0.005952381
C5.0	model	rules
	winnow	FALSE
	trials	67
KNN	k	27
Naïve Bayes	fL	0
	usekernel	FALSE
	adjust	1
Random Forest	mtry	12
Support Vector Machine	sigma	0.1786673
	C	581.883

Evidence demonstrated that the class imbalance (i.e., unequal size of the dependent variable), which is just the situation in our sample, can substantially impact the performance of machine learning [28]. We therefore adopted synthetic minority over-sampling technique by under-sampling the adequate class and over-sampling the inadequate class to improve the model performance [28]. A widely held view remains that it is better to test a given model with samples that were not used for training when building a predictive model [29, 30]. We therefore adopted the holdout method by randomly splitting the data into 70% for training and 30% for testing model [29, 31] in order to better estimate the model’s accuracy and to avoid any possible overfitting problem. Further, 10-fold cross validation method with three repeats, which has be viewed as the de facto standard for estimating model performance [32], was applied to all seven classifiers with the training dataset. More specifically, the data set is partitioned into ten subsets of roughly equal size, wherein any nine subsets were used for model training, and the remaining one subset was used for model testing and estimating model performance. The above procedures were repeated for three times. Finally, overall model performance was calculated by averaging model performances each time [32].

### Performance measures

We adopted the accuracy, area under receiver operating characteristic curve (AUC), sensitivity, specificity, and the kappa statistic for assessing the model performance. The accuracy is defined as the ratio of dataset records that are correctly classified [30]. The sensitivity refers to the percentage of positive records that are correctly classified, while the specificity measures the proportion of negative records that are correctly identified [30]. Further, by plotting the sensitivity against (1 – specificity), the performance of a binary classifier can be assessed via AUC [33]. An AUC value of at least 0.8 is considered as good performance, while at least 0.9 is excellent [33]. Finally, the kappa, originally used to assess the agreement between two raters [27], adjusts accuracy by accounting for the probability of a correct prediction by chance alone [32]. A kappa value ranges between 0.4 and 0.6 indicates moderate agreement, 0.6–0.8 as substantial agreement, and 0.8–1 as almost perfect agreement [34].

## Results

### Data profiles

Table 3 shows the predictors and descriptive statistics for schizophrenic patients with and without pneumonia. There were 106 in-patient cases with and 79 in-patients without hospital-acquired pneumonia.

**Table 3:** Descriptive statistics for patients with/without pneumonia

Variable	Patients with pneumonia (*n* = 106)		Patients without pneumonia (*n* = 79)	
	Range	Summary statistics	Range	Summary statistics
Gender	Male/Female	Male: 77, Female: 29	Male/Female	Male: 53, Female: 26
Age	26~82	M = 52.51, SD = 12.33	22~81	M = 51.28, SD = 14.66
Clozapine use	Yes/No	Yes = 50, No = 56	Yes/No	Yes = 5, No = 74
Drug-drug interaction	Yes/No	Yes = 15, No = 91	Yes/No	Yes = 1, No = 78
Coughing	Yes/No	Yes = 29, No = 77	Yes/No	Yes = 16, No = 63
Dosage	0~800	M = 113.42, SD = 161.09	0~350	M = 10.13, SD = 47.62
Duration of medication	0~377	M = 47.93, SD = 82.41	0~295	M = 8.61, SD = 42.77
Change of leukocyte count	− 5200~5021	M = 136.60, SD = 1896.11	− 8840~5500	M = 658.63, SD = 1996.90
Change of neutrophil count	−29.4~27.3	M = −1.04, SD = 9.16	−18.6~32.6	M = 3.84, SD = 9.35
Change of blood sugar level	− 318~153	M = −0.15, SD = 40.12	− 49~133	M = 0.29, SD = 21.52
Change of weight	−16.5~12.5	M = 0.82, SD = 3.96	−11.5~13	M = 1.05, SD = 4.53

### Model performances

Table 4 demonstrates the results of seven specific machine learning algorithms. Accuracy, AUC, sensitivity, specificity, and kappa were used to assess the performance of those seven methods. Since we adopted ten-fold cross validation for estimating model performance, the means and standard deviations of the above five metrics can be calculated for the training sample.

**Table 4:** Performance evaluation of models employed

Sample	Method	Accuracy(SD)	AUC(SD)	Sensitivity(SD)	Specificity(SD)	Kappa(SD)
Train	CART	0.804(0.089)	0.851(0.074)	0.739(0.094)	0.851(0.106)	0.597(0.180)
	C5.0	0.912(0.033)	0.971(0.018)	0.868(0.047)	0.942(0.030)	0.819(0.068)
	KNN	0.645(0.083)	0.696(0.066)	0.628(0.127)	0.657(0.130)	0.282(0.162)
	NB	0.675(0.095)	0.798(0.094)	0.868(0.096)	0.544(0.098)	0.376(0.178)
	RF	0.917(0.017)	0.971(0.016)	0.891(0.048)	0.937(0.032)	0.831(0.035)
	SVM	0.871(0.030)	0.936(0.030)	0.832(0.077)	0.923(0.043)	0.733(0.062)
	LGR	0.670(0.084)	0.762(0.083)	0.621(0.076)	0.706(0.139)	0.330(0.160)
Test	CART	0.830	0.880	0.904	0.732	0.648
	C5.0	0.945	0.993	0.989	0.887	0.887
	KNN	0.667	0.701	0.745	0.563	0.312
	NB	0.733	0.831	0.628	0.873	0.479
	RF	0.927	0.994	1.000	0.831	0.849
	SVM	0.897	0.953	0.968	0.803	0.786
	LGR	0.739	0.823	0.798	0.662	0.464

Note: SD denotes standard deviation

Among the seven methods employed, RF has the highest accuracy rate (0.917), followed by C5.0 (0.912), SVM (0.871), and CART (0.804). The remaining classifiers had an accuracy rate of less than 0.7. In terms of AUC, RF, C5.0, and SVM have an AUC value of higher than 0.9, indicating excellent classifier performance. The AUC values of CART are higher than 0.8, demonstrating good performance. Further, NB and LGR have fair performance, while KNN has poor performance in terms of AUC. In sum, C5.0, RF, SVM, and CART perform well among the seven classifiers for the training dataset. Further, the kappa value of RF and C5.0 is 0.831 and 0.819, respectively, indicating almost perfect agreement. The performance of SVM is substantial, while CART is moderate based on their kappa statistics. The remaining classifiers performed poorly.

To prevent overfitting, we further predicted our models with testing dataset. C5.0, RF, SVM, and CART still perform better than the remaining methods in terms of accuracy (*see* Table 4). Specifically, C5.0 and RF have an accuracy rate higher than 0.9, while CART and SVM have an accuracy rate higher than 0.8. In terms of AUC, the performance of C5.0, RF, and SVM belongs to excellent, while CART belongs to good. The kappa statistics also demonstrate the same results.

A comprehensive assessment of the various performance metrics reveals that RF, C5.0, and SVM perform better than CART among better classifiers. KNN has the poorest performance. Further, no sign of overfitting among the other seven classifiers exists since their accuracy rate, AUC, and kappa statistics of testing dataset are higher than that of training dataset.

### Predictor importance

Besides contrasting the performance of various models, we also ranked the predictor importance based on information gain and gain ratio [29]. The gain ration is a bias-corrected criterion based on information gain, and both criteria are often utilized for classifying algorithms [29]. The top six important predictors are the same based on both criteria, only the order of variables is different. As Table 5 shows, the top six imperative predictors influencing acquiring pneumonia for schizophrenia in-patients were dosage, clozapine use, duration of medication, change of neutrophil count, change of leukocyte count, and drug-drug interaction.

**Table 5:** Ranking of investigated variables according to gain ratios

Variables	Information gain	Ranking	Gain ratio	Ranking
Dosage	0.124	1	0.217	1
Clozapine use	0.093	2	0.150	2
Duration of medication	0.072	3	0.115	3
Change of neutrophil count	0.046	4	0.068	6
Change of leukocyte count	0.039	5	0.107	4
Drug-drug interaction	0.028	6	0.076	5

## Discussion

With appropriate treatment, schizophrenia can be well-controlled via medications and psychological support [3]. However, schizophrenic patients are susceptible to pneumonia due to anti-psychotic medications [5, 12]. It is therefore particularly important to better understand risk factors of pneumonia that accompany the treatment of schizophrenia. Despite a number of studies have investigating risk factors of pneumonia for schizophrenia [5, 6], literature however revealed that little of those studies which adopted machine learning techniques for prediction. Among the seven adopted machine learning algorithms in our study, RF and C5.0 exhibited the optimal prediction accuracy rather than the remaining algorithms.

In accordance with information gain and gain ratio, we also identified and ranked the top six crucial risk factors including dosage, clozapine use, duration of medication, change of neutrophil count, change of leukocyte count, and drug-drug interaction. Among them, clozapine dosage, clozapine prescription, and prescription duration were major factors said to have influenced the occurrence of pneumonia. Knol, Van Marum, Jansen, Souverein, Schobben and Egberts [6] found that pneumonia risk was the highest during the first week after initiation of an anti-psychotic medication. In addition, use of typical anti-psychotic (i.e., clozapine) and titration of dosage was also associated with a greater risk of pneumonia [5]. Therefore, physicians should be careful about setting an appropriate dosage and the duration of medication when prescribing anti-psychotic medications. Further, we also found that the changing of leukocyte and neutrophil count might be indicators affecting the development of pneumonia among schizophrenic patients. Clozapine is notorious for its dangerous adverse effects, for example, neutropenia and agranulocytosis [35]. Because the immuno-compromised status would decrease the protection against bacteria invasion and transmission in human bodies, physicians should therefore closely monitor leukocyte and neutrophil count in hospitalized schizophrenic patients. Finally, drug-drug interaction is also confirmed as a risk factor for pneumonia when treating schizophrenic patients. Hematological adverse effects including neutropenia and agranulocytosis are enhanced when clozapine was prescribed simultaneously with anxiolytic, anti-convulsant, antimicrobials, proton pump inhibitors and other gastro-intestinal agents [36]. Physicians are therefore advised to pay close attention to plausible drug-drug interaction among differing drugs whenever prescribing anti-psychotic drugs.

Although the outcome variables and performance metrics are not entirely consistent, it is still worthwhile to make a comparison and contrast between our study and prior pneumonia-related studies that had adopted machine-learning approaches. In their study to predict pneumonia patients’ readmission, Hilbert, Zasadil, Keyser and Peele [23] adopted a decision tree learner and found that age and gender are considered as important factors which were not determined in our study. The AUCs of CART and C5.0 derived from our proposed models (0.880 and 0.993) are both higher than that of Hilbert, Zasadil, Keyser and Peele [23] (0.658). By employing k-nearest neighbor method, Chen, Huang, Tan, Chang and Chang [24] built an abnormal lung sounds diagnostic model with an error rate of 6.8%. The k-nearest neighbor method however revealed the poorest performance in terms of all of the performance metrics found in our study. Khajehali and Alizadeh [25] adopted a boosting naïve Bayes learner for predicting pneumonia patients’ length-of-stay with their model showing a better than average prediction accuracy of 95.2%. Their model outperformed our model which utilized non-boosting naïve Bayes with a prediction accuracy of 67.5%. Boosting, one of the ensemble methods, is known to improve the performance of a predictive model [32]. Caruana, Lou, Gehrke, Koch, Sturm and Elhadad [19] adopted a random forest learner and logistic regression to predict pneumonia risk considerate of from 46 features. The resulting AUCs for random forest and logistic regression were 0.846 and 0.843, respectively. In our study, random forest, a widely recognized learner with well-performance across a broad range of problems [37], outperformed the study of Caruana, Lou, Gehrke, Koch, Sturm and Elhadad [19], but the logistic regression in our model performed poorer than that of Caruana, Lou, Gehrke, Koch, Sturm and Elhadad [19]. Huang, Chen and Hsu [26] constructed a clinical decision model via a support vector machine learner for predicting pneumonia readmission, and their model achieved 83.9% predictive accuracy with six predictors, including age, gender, number of medication, length of admission, number of comorbidities, and total admission cost. Our proposed model utilizing a support vector machine learner provided a slightly higher predictive accuracy (87.1%) than that of Huang, Chen and Hsu [26].

Our study is one of the few, to our knowledge, that adopted a machine learning approach in predicting the risk factors of acquiring pneumonia specifically among schizophrenic patients. By utilizing machine learning techniques, risk factors commonly neglected by traditional statistical models can be discovered. Further, by utilizing seven differing classifiers, the findings can be contrasted and compared in order to select the best predictive model for helping preventing schizophrenic patients from the occurrence of pneumonia. Several academic and practical implications can be obtained from our findings.

Our study adopted classification and regression tree, decision tree, k-nearest neighbors, naïve Bayes, random forest, support vector machine, and logistic regression and compared the performance of these classifiers. Random forest, decision tree, and support vector machine outperformed the remaining classifiers. The results demonstrates that machine learning techniques have a high potential for predicting risk factors among hospitalized schizophrenic patients for acquiring pneumonia. Further, the plausible negative effects of class imbalance, a common situation for health data, seemed to be diminished in our model by employing synthetic minority over-sampling technique. Future studies can utilize this technique to neutralize the class imbalance issue.

The potential risks factors utilized by our model are based on clinical evidence, and our model further identified six crucial risk factors predicting schizophrenic patients who may acquire pneumonia. The findings of our study can be utilized as an important reference for psychiatrists by reminding them to pay closer attention to these risk factors whenever diagnosing and then treating schizophrenic patients. One step further, our developed predictive model may be integrated into a CPOE. Psychiatrists can acquire a timely alert regarding the possibility of the onset of pneumonia when they use a CPOE to diagnose and treat schizophrenia in-patients. With information about potential pneumonia risk, early prevention and intervention procedures and decision plans can be formulated in combination with related clinical experiences.

Several limitations should be noted in our study. First, the analyzed cases were extracted from a small-scale hospital, the generalizability of our findings can be limited. Future studies are suggested to gather more cases from a wider variety of hospitals. Secondly, other potential risk factors which were unavailable from the review of medical records can be considered for use in the model.

## Conclusions

The purpose of our study is to build a model for predicting the risk factors of pneumonia among patients with schizophrenia. To achieve this goal, we employed seven machine learning algorithms to build classification models for prediction. 185 eligible cases were used to validate the prediction accuracy of the adopted techniques. Overall, RF and C5.0 demonstrated optimal performance among those adopted machine learning techniques. Further, our model also identified the top six risk factors for pneumonia among patients with schizophrenia.

## Abbreviations

AUC: Area under Receiver Operating Characteristic Curve
C5.0: C5.0 Decision Tree
CART: Classification and Regression Tree
CPOE: Computerized physician order entry
ICD-9-CM: International Classification of Diseases, Ninth Revision, Clinical Modification
KNN: K-Nearest Neighbors
LGR: Logistic Regression
NB: Naïve Bayes
RF: Random Forest
SD: Standard Deviation
SVM: Support Vector Machine

## References

[1] Birnbaum HG, Morley M, Greenberg PE, Cifaldi M, Colice GL (2001). Economic burden of pneumonia in an employed population. Arch Intern Med.

[2] Mortensen EM, Coley CM, Singer DE, Marrie TJ, Obrosky DS, Kapoor WN, Fine MJ (2002). Causes of death for patients with community-acquired pneumonia: results from the pneumonia patient outcomes research team cohort study. Arch Intern Med.

[3] World Health Organization. Schizophrenia. 2018. http://www.who.int/en/news-room/fact-sheets/detail/schizophrenia. Accessed 3 Sept 2018.

[4] Nicholl D, Akhras KS, Diels J, Schadrack J (2010). Burden of schizophrenia in recently diagnosed patients: healthcare utilisation and cost perspective. Curr Med Res Opin.

[5] Kuo CJ, Yang SY, Liao YT, Chen WJ, Lee WC, Shau WY, Chang YT, Tsai SY, Chen CC (2013). Second-generation antipsychotic medications and risk of pneumonia in schizophrenia. Schizophrenia Bull.

[6] Knol W, Van Marum RJ, Jansen PAF, Souverein PC, Schobben AFAM, Egberts ACG (2008). Antipsychotic drug use and risk of pneumonia in elderly people. J Am Geriatr Soc.

[7] Gupta S, Boville BM, Blanton R, Lukasiewicz G, Wincek J, Bai C, Forbes ML (2015). A multicentered prospective analysis of diagnosis, risk factors, and outcomes associated with pediatric ventilator-associated pneumonia. Pediatr Crit Care Me.

[8] Manabe T, Teramoto S, Tamiya N, Okochi J, Hizawa N (2015). Risk factors for aspiration pneumonia in older adults. PLoS One.

[9] Cooper GF, Aliferis CF, Ambrosino R, Aronis J, Buchanan BG, Caruana R, Fine MJ, Glymour C, Gordon G, Hanusa BH (1997). An evaluation of machine-learning methods for predicting pneumonia mortality. Artif Intell Med.

[10] Heckerling PS, Gerber BS, Tape TG, Wigton RS (2004). Use of genetic algorithms for neural networks to predict community-acquired pneumonia. Artif Intell Med.

[11] Kim SY, Diggans J, Pankratz D, Huang J, Pagan M, Sindy N, Tom E, Anderson J, Choi Y, Lynch DA (2015). Classification of usual interstitial pneumonia in patients with interstitial lung disease: assessment of a machine learning approach using high-dimensional transcriptional data. Lancet Resp Med.

[12] Hung GCL, Liu HC, Yang SY, Pan CH, Liao YT, Chen CC, Kuo CJ (2016). Antipsychotic reexposure and recurrent pneumonia in schizophrenia: a nested case-control study. J Clin Psychiatry.

[13] Tatro DS (2015). Drug interaction facts 2015 : the authority on drug interactions.

[14] Trifirò G, Gambassi G, Sen EF (2010). Association of community-acquired pneumonia with antipsychotic drug use in elderly patients: a nested case–control study. Ann Intern Med.

[15] Leo RJ, Kreeger JL, Kim KY, Dalmady-Israel C, Mailhot C (1996). Cardiomyopathy associated with clozapine. Ann Pharmacother.

[16] Dunk LR, Annan LJ, Andrews CD (2006). Rechallenge with clozapine following leucopenia or neutropenia during previous therapy. Brit J Psychiat.

[17] Newcomer JW, Haupt DW, Fucetola R (2002). Abnormalities in glucose regulation during antipsychotic treatment of schizophrenia. Arch Gen Psychiat.

[18] Chapman WW, Fizman M, Chapman BE, Haug PJ (2001). A comparison of classification algorithms to automatically identify chest x-ray reports that support pneumonia. J Biomed Inform.

[19] Caruana R, Lou Y, Gehrke J, Koch P, Sturm M, Elhadad N (2015). Intelligible models for healthcare: predicting pneumonia risk and hospital 30-day readmission. Proceedings of the 21th ACM SIGKDD international conference on knowledge discovery and data mining.

[20] Worster A, Haines T (2004). Advanced statistics: understanding medical record review (mrr) studies. Acad Emerg Med.

[21] Gearing RE, Mian IA, Barber J, Ickowicz A (2006). A methodology for conducting retrospective chart review research in child and adolescent psychiatry. Can Acad Child Ad Psychiat.

[22] R Core Team. R: a language and environment for statistical computing. Vienna, Austria: R Foundation for Statistical Computing. 2018. https://www.R-project.org/. Accessed 9 November 2018.

[23] Hilbert JP, Zasadil S, Keyser DJ, Peele PB (2014). Using decision trees to manage hospital readmission risk for acute myocardial infarction, heart failure, and pneumonia. Appl Health Econ Health Policy.

[24] Chen CH, Huang WT, Tan TH, Chang CC, Chang YJ. Using k-nearest neighbor classification to diagnose abnormal lung sounds. Sensors. 2015;15(6). 10.3390/s150613132. 10.3390/s150613132 PMC4507578 26053756

[25] Khajehali N, Alizadeh S (2017). Extract critical factors affecting the length of hospital stay of pneumonia patient by data mining (case study: an iranian hospital). Int J Med Inform.

[26] Huang JS, Chen YF, Hsu JC (2014). Design of a clinical decision support model for predicting pneumonia readmission. In: 2014 international symposium on computer, consumer and control: 10–12 June 2014 2014.

[27] Kuhn M. Building predictive models in r using the caret package. J Stat Softw. 2008;28(5). 10.18637/jss.v028.i05.

[28] Chawla NV, Bowyer KW, Hall LO, Kegelmeyer WP (2002). Smote: synthetic minority over-sampling technique. J Artif Intell Res.

[29] Kuhn M, Johnson K (2013). Applied predictive modeling.

[30] Provost F, Fawcett T (2013). Data science for business: What you need to know about data mining and data-analytic thinking.

[31] Raschka S. Model evaluation, model selection, and algorithm selection in machine learning 2018. https://arxiv.org/abs/1811.12808v2. Accessed 19 Jan 2019.

[32] Lantz B (2015). Machine learning with r.

[33] Hosmer DW, Lemeshow S, Sturdivant RX (2013). Applied logistic regression.

[34] Landis JR, Koch GG (1977). The measurement of observer agreement for categorical data. Biometrics..

[35] Citrome L, McEvoy JP, Saklad SR (2016). Guide to the management of clozapine-related tolerability and safety concerns. Clin Schizophr Relat Psychoses.

[36] Jeon SW, Kim YK (2017). Unresolved issues for utilization of atypical antipsychotics in schizophrenia: antipsychotic polypharmacy and metabolic syndrome. Int J Mol Sci.

[37] Caruana R, Karampatziakis N, Yessenalina A (2008). An empirical evaluation of supervised learning in high dimensions. Proceedings of the 25th international conference on machine learning.

